# Topics of Public Interest

**Published:** 1909-01

**Authors:** 


### TOPICS OF PUBLIC INTEREST.

### State Board of Medical Examiners hold Court.

**Medical Society of the County of New York Seeks to Revoke Licenses—Hearing Before a Committee of the State Board of Medical Examiners.**

Six physicians, convicted of various felonies and misdemeanors, were given a hearing Friday, December 18, 1908, before three members of the State Board of Medical Examiners in proceedings brought by the Medical Society of the County of New York for the revocation of their licenses. This action is the first to be brought under the medical law passed in 1907, and is likewise the first one that the County Medical Society has yet instituted in its 102 years of existence. These are the physicians placed on trial in the disbarment proceedings:

Dr. Moses Jackson, the former Coroner, found guilty of bribery on February 2, 1905, judgment on whom was suspended by Judge Foster. Jackson was registered to practise in the county in 1884.

Edward E. Conrad, convicted on April 8, 1904, before Recorder Goff of attempted abortion and sentenced to an indeterminate sentence of from one to two years in state prison. Conrad did his time. He was admitted to practise September 14, 1887, upon the presentation of a diploma from the Chaddock School of Medicine of Quincy, Ill., dated 1889, and indorsed by the Bellevue Hospital Medical College, September 14, of the same year.

Dr. Frank J. Dudenhausen, found guilty of practising under the assumed name of old Dr. Dale and sentenced as a second offender on May 27, 1908, to six years and five months in state prison. Dudenhausen is now out on bail pending an appeal. He was licensed by the Regents to practise in this State on April 15, 1893, and was registered in the county of New York in April, 1902.

Dr. Francis Gray Blinn, sentenced April 23, 1908, for attempted abortion. He is now serving from one to two years in Sing Sing. Blinn got his diploma from the Hahnemann Medical College of Chicago in 1885, and after receiving the indorsement of the New York Homeopathic Medical College and hospital, he was registered to practise in the county in 1890.

Dr. Rudolph Mielke, who worked for the Collins Medical Institute, was convicted of sending indecent literature through the mails and was fined $50 by the Court of Special Sessions on July 18, 1907. Mielke was registered in 1880.

Dr. Robert Ormsby was fined $100 by the Court of Special Sessions for selling drugs for criminal purposes. Ormsby was registered in 1880.

These six men are being prosecuted under Chapter 344, section 11, of the laws of 1907. This among other things provides that a practitioner "who is guilty of a crime or misdemeanor" may have his license to practise in the State revoked or his registration to practise in the county annulled or may suffer the double penalty at the option of the Regents. The first four named come under the jurisdiction of the old law, which prescribed this punishment only against those physicians guilty of a felony. While no physician is supposed to practise after he has been convicted of lawbreaking, it is very hard to prove that they do; whereas if their license be revoked or their registration annulled they may be punished for the mere attempt to set up in business again. For this reason the County Medical Society applied for the revocation of the licenses, so that if the six men should try to carry on their vocation any longer a case could be proved against them more easily.

The three members of the Board of Medical Examiners appointed to look into the County Medical Society's application for the revocation of licenses were Dr. William Warren Potter of Buffalo, Chairman; Dr. Lee H. Smith of Buffalo and Dr. Floyd M. Crandall of New York.

The committee met in the office of Dr. Maurice J. Lewi, secretary to the board. Frank B. Gilbert, chief of the law division of the State Department of Education, appeared as legal adviser to the committee. The county medical society was represented by its counsel, Almuth C. Vandiver of the firm of Whitman & Vandiver of 32 Nassau street, New York. Dr. Augustus S. Downing, assistant commissioner of education, was also present.

Most of the accused had little to offer in the way of defence. The only question of law that was raised was brought up by Philip Carpenter, who appeared for Jackson. Mr. Carpenter maintained that under the decision rendered in October by the Court of Appeals in the case of the People vs. Fabian, Jackson had not been convicted within the meaning of the law, as the sentence upon him had not yet been pronounced. He maintained therefore that the committee had no jurisdiction over his client.

Mr. Vandiver insisted that in the Fabian case, which was one of election frauds, the Appellate Division construed the word conviction strictly, as it was used in the State Constitution and in the election law, and that the decision does not therefore apply to Jackson's case, as the statute under consideration contemplates only the guilt of the accused. This is shown, he declared, by the wording of the law. Mr. Carpenter got thirty days to file his brief. The question involved in Jackson's case may be taken up to the Supreme Court of the United States.

Thomas S. Morgan obtained for his client, Dudenhousen, the assurance from the board that they would withhold their recommendation in this case till January, when Dudenhausen's application for a retrial will be argued. Dudenhausen is now out on a certificate of reasonable doubt.

Decision in Blinn's case will also be withheld till the February term of the Appellate Division, when, as Amos H. Stevens, Blinn's attorney, told the committee, argument on an appeal will be made.

Mielke was defended by Dudley Field Maloney, representing George Gordon Battle, and by George A. Knoblock, representing John J Vause. Mielke's counsel took exception to the presence of Dr. Crandall on the committee, on the ground that he was a member of the County Medical Society, at whose instance the prosecution was begun. The objection was overruled. Then they asked for an adjournment, which was denied. The defence, however, was given an opportunity to submit an application to be permitted to present oral testimony to the board. Mielke pleaded guilty when he was on trial in 1907.

Conrad was represented by Judge W. M. K. Olcott, who asked postponement of action, pending application to the governor for pardon. Granted.

The committee will probably not make its findings known much before next March, as the Regents do not meet until then.

The Regents will take final action on the findings and recommendations of the committee. The accused may either have their registration annulled, their license revoked, or may be suspended from practice. If their license is revoked, they may apply again after a year for its renewal.

---

### Dr. H. W. Wiley Visits Buffalo.

**The author of the Pure Food and Drug Law on building a conscience—Average business-man was defective in this regard before pure food law, says Dr. Wiley Concerning Food—No such thing as Brain Foods or Nerve Foods, declares the Federal Expert.**

Dr. H. W. Wiley, chief of the bureau of chemistry in the Department of Agriculture, who was in a sense, the author of the national pure food and drug law, spoke before the Westminster Club on Tuesday evening, December 8, 1908. He rather opened the eyes of Buffalonians on what the pure food and drug law is doing.

The conscience of the average American business man, said Dr. Wiley, isn't just calloused or seared—it's a minus quantity. But we have discovered a conscience builder. The average business men can build up a conscience on the strength of a few fines in the federal courts. Conscience can be made to sprout, and we're sprouting a few every day.

Don't be misled by foodists. Some claim one kind of food is the best and others, another. There is no such thing as a brain food, or a nerve food or a skin food. Because one dealer advertised a certain article as brain food, a federal judge handed him the limit of a sentence. I was put on the stand in that investigation as an expert. When asked if the article would not nourish the brain, I responded that it would also act as a great stimulus to the big toe.

So don't be misled by brain foods. You won't get mentally strong by eating brain foods, though you may get dyspepsia.

The national pure food law when passed was thought by Congress to be a joke; it hasn't proved so. Out of 200 cases we have tried, we have convicted all but two, so if a man gets the pure food inspectors after him he can figure his chances at about 1 to 100.

The pure food law has two evils to remedy: false representation of the nature of the substance (i.e. misleading), and debased or adulterated articles. The law applies to foods, drink and drugs for men as well as animals.

Under the pure food law you can't feed Fido adulterated food; you can't feed birdie seeds that are poisonous or we'll be after you.

Those who fought the measure the hardest are now its strongest supporters. American made wines with French labels are bygones; maple sugar that isn't—isn't now, unless it says so on the sugar. It's a guarantee of American goods and is overriding the idea that 'the American people love to be humbugged.'

Dr. Wiley was entertained at luncheon the afternoon before the lecture, at the plant of the Shredded Wheat Company at Niagara Falls. Among those present were. Dr. Samuel Van Vranken Holmes, Dr. Hamilton W. Benham, Dr. F. Whitehill Hinkel, George R. Howard, Loran L. Lewis, Jr., and S. N. McWilliams of Buffalo, and president A. J. Porter, general manager M. B. Butler; secretary, H. W. McBean and F. A. DeWeese, director of publicity of the Shredded Wheat Company.

---

### The Fight Against Consumption.

**Buffalo enlisted for the war against Consumption—Fight against the White Plague will be carried on with greater vigor—Two hospitals necessary—One for Contagious Diseases and one for Tuberculosis are equally needed.**

STEPS were taken December 10, 1908, at a largely attended meeting of representatives of the various charitable and relief associations of the city to form what will be known as the Buffalo Association for the Relief and Control of Tuberculosis. The meeting was held at the Hotel Iroquois with Dr. Matthew D. Mann as chairman and Irving S. Underhill secretary of the meeting.

Among those present were the Mayor, J. N. Adam, Dr. De Lancey Rochester, Dr. John H. Pryor, the Reverend Cameron J. Davis, the Reverend Robert Freeman, Walter L. Brown, Dr. Francis E. Fronczak, Frederic Almy, Hugh Kennedy, Ansley Wilcox, Dr. F. Park Lewis and Dr. Gustav A. Pohl. There were also about a score of the city's philanthropic women at the meeting.

### MUST ISOLATE KNOWN CASES.

At the outset, Dr. Pryor stated briefly the object of the gathering. He said that last year 554 persons died of tuberculosis in this city. "It has been found," continued Dr. Pryor, "that there are five times as many cases of consumption as there are deaths. Therefore, multiplying 554 by five, we have 2,770 people suffering today in their homes with this disease and infecting others because Buffalo has no facilities for isolating those afflicted with the malady. There are but 60 beds for consumptives in this city. Experience has shown that the only way to fight tuberculosis is on a large scale. We need a municipal hospital for tuberculosis, and the purpose of this new association is to promote that end."

Mr. Ansley Wilcox then expressed his sympathy with the aims of the proposed association and his belief that both a contagious disease and a tuberculosis hospital should be established in this city. The chairman then called for a vote on the question: Shall the chairman be given authority to name a committee of fifteen to consider the organisation of an association to fight tuberculosis? the committee to report at a future meeting. This was carried by a viva voce vote. Later, an amendment to the effect that the committee have power to add to their number was also carried.

### TWO HOSPITALS NEEDED.

Another action taken by the meeting will, doubtless, create much favorable comment. Dr. Pryor asked that the meeting go on record as in favor, not alone of a hospital for the treatment of tuberculosis, but also of a municipal hospital for contagious diseases. Dr. De Lancey Rochester, in seconding the motion, expressed his pleasure that such action would forever quash the rumor that those in favor of the tuberculosis hospital were either against or apathetic toward the movement for a hospital for contagious diseases. "We need not only one, but both, hospitals," declared Dr. Rochester.

Secretary Underhill read a list of the organisations which had been asked to send delegates to yesterday's meeting, numbering nearly twenty of various kinds,—religious, medical, social, musical, business and philanthropic.

### THE FIGHT IN NEW YORK CITY.

New York City is already stirred up in a fight against tuberculosis. Observers say it is the greatest crusade waged in the history of the nation. There are church mass meetings, labor mass meetings, social workers' mass meetings and nurses' meetings, and on a recent Sunday, every clergyman in New York was asked to say something from his pulpit about the battle for the consumptive.

At the outset of the conference being held in the American Museum of Natural History, $60,000 was raised to pay for installing the tuberculosis exhibit brought from the international congress recently held in Washington. This exhibit, which fills the museum, includes all states in this country and the foreign countries of Europe.

About 40,000 posters giving facts about tuberculosis have been printed. One of the large posters states that every three minutes someone dies from consumption in the United States: that 10,000 people died in New York last year from the disease, and that 16,567 persons died in the last year in this state.

In all the surface, subway and elevated cars in New York, a card with this motto has been placed:

We must care for the consumptive in the right place, in the right way and at the right time until he is well. Not in the wrong place, in the wrong way and at the wrong time until he is dead.

Record crowds have been visiting the great tuberculosis exhibit in the American Museum of Natural History. During the first six days of the conference 140,000 people visited it. The climax was reached Sunday, December 6, when 40,000 people were admitted to the museum between 1 and 5 o'clock. So great was the crush, that the police reserves had to be called out.

Monday, December 21, was known as New York State day. It was held under the auspices of the State Charities Aid Association and the New York State department of health. Among the speakers on that occasion were Dr. Herman M. Biggs, Dr. Eugene H. Porter, State Commissioner of Health, John Williams and Dr. John H. Pryor of Buffalo.

